# Preliminary Experience with Transdermal Preoperative Hormonal Treatment Before Severe Hypospadias Repair: Synergy Between Pediatric Surgeons and Endocrinologists

**DOI:** 10.3390/children12030296

**Published:** 2025-02-27

**Authors:** Laura Lucaccioni, Filippo Ghidini, Paolo Repetto, Grazia Spampinato, Anna Insalaco, Sara Vandelli, Viola Trevisani, Lorenzo Iughetti, Pier Luca Ceccarelli

**Affiliations:** 1 Pediatric Unit, Department of Medical and Surgical Sciences of the Mother, Children and Adults, University of Modena and Reggio Emilia, 41125 Modena, Italy; 2 Pediatric Surgery Unit, Department of Medical and Surgical Sciences of the Mother, Children and Adults, University of Modena and Reggio Emilia, 41125 Modena, Italy; 3 Post-Graduate School of Pediatrics, University of Modena and Reggio Emilia, 41125 Modena, Italy; 4 PhD Program, Clinical and Experimental Medicine, University of Modena and Reggio Emilia, 41125 Modena, Italy

**Keywords:** hypospadias, preoperative hormonal treatment, testosterone, transdermal testosterone, urology, endocrinology

## Abstract

**Objectives:** The preoperative hormonal treatment (PHT) in eligible patients has the potential to become an asset for the treatment of severe hypospadias. The aim of the paper is to report the preliminary results on tolerability and efficacy of tailored transdermal PHT with testosterone before primary hypospadias repair, resulting by the joint activity between pediatric surgeons and endocrinologists. **Methods:** A retrospective study collected all the patients affected by severe hypospadias, with a glans width (GW) < 14 mm and/or a penile ventral curvature > 30°, treated with preoperative transdermal testosterone gel 2% at a standard dose of 2 mg/day after pediatric endocrinologist evaluation, from December 2020 to February 2024. Increases in GWs and penile lengths (PLs), together with adverse events and the rate of surgical complications, were reported. **Results:** During the period, ten patients were included and received transdermal PHT for 43 (±15) days on average. The treatment with PHT stopped 52 (±23) days before surgery. PL increased 0.76 (±0.27) cm (+37%) on average, and GW increased 0.42 (±0.26) cm (+40%). No adverse events were described. Three surgical complications were reported with an overall rate of 30%. **Conclusions:** Transdermal PHT was well-tolerated and showed a positive impact on the treatment of severe hypospadias. Future investigations might confirm these findings.

## 1. Introduction

Severe proximal hypospadias repair still challenges surgeons due to the high risk of complications. Several aspects have been investigated in an attempt to improve the outcomes of this surgery [1].

Preoperative hormone treatment (PHT) was introduced in the early 1970s to enlarge penile size and improve tissue trophism prior to the surgical repair [2]. However, the rationale for PHT has been emphasized in the last two decades. Firstly, more than half of the severe hypospadias cases presented with a small glans [3]. Secondly, a glans width (GW) less than 14 mm has been recognized as an independent negative risk factor [4]. Finally, in these selected patients, PHT increased GW by more than two standard deviations [5]. For these reasons, PHT might be considered a useful strategy to deal with severe hypospadias.

On the other hand, the actual efficacy of PHT is controversial due to a higher rate of surgical complications [6,7], possible unresponsiveness to hormonal stimulations due to endocrinological reasons (such as androgen insensitivity syndrome) that are often not checked before surgery, and the invasiveness of repeated intramuscular injections in infants. Recently, several studies investigated the ratio of surgical complications in patients with hypospadias who have and have not undergone PHT in order to ascertain whether the treatment is beneficial to the surgeon during the operation or to the patient in reducing complication rates. A key challenge in assessing complication rates is the stratification of surgical outcomes based on the severity of hypospadias and the age at surgery [8,9]. In a large cohort of distal hypospadias, Godlewski et al. demonstrated a reduction in post-operative complications with the use of PHT, after correction for age at surgery, preoperative glans width, preoperative testosterone status, and urethroplasty length [10]. A recent study by Jackson et al. also showed a complication rate of 4/22 patients who received topical androgen prior to initial hypospadias surgery and had completed all surgical stages [11]. It is imperative to recognize that the severity of hypospadias is directly related to the likelihood of post-operative complications. We report our initial experience with tailored PHT after endocrinological evaluation to demonstrate its good tolerability and efficacy in improving penile tissue trophism in patients with severe hypospadias.

## 2. Materials and Methods

The design of this study was retrospective and single-centered, in a time span ranging from December 2020 to February 2024.

The transdermal PHT (Tostrex^®^, Kyowa Kirin S.r.l. Modena, Italy) was proposed to the patients affected by proximal or midshaft hypospadias, according to the following criteria: GW inferior to 14 mm and/or clinically relevant penile ventral curvature (superior to 30°) [4,12]. All the patients that underwent transdermal PHT were enrolled in this study.

A multidisciplinary preoperative consultation, including pediatric endocrinological and surgical assessment, was performed to propose the PHT. Ano-genital distances (AGD) and penile measurements (GW and penile length (PL)) were measured using a vernier caliper by the same operator for each patient, with the children in the supine position with hips flexed. AGD measurements were taken from the center of the anus to a genital landmark as (1) the anterior base of the penis where the penile tissue meets the pubic bone (AGD-AP) and (2) the base of the scrotum where the skin changes from rugate to smooth (AGD-AS). For PL, the length of the ventral penis, while flaccid, was measured from the base to the glans. GW was assessed by measuring the diameter of the glans, uncovered by the foreskin. Testicular volume and position were also detected. Moreover, anthropometrics such as weight, length, and head circumference were assessed according to WHO standards.

A standard karyotype was obtained for each patient. Adrenal and minipubertal assessment, testosterone (T), dihydrotestosterone (DHT), and anti-Mullerian hormone (AMH) were performed when possible. The enzyme immunoassay method was used for the direct quantitative determination of the above hormones in serum by the same hospital laboratory.

Once the endocrinological cause of hypospadias had been assessed, after the legal guardians’ consent, topical testosterone gel (2%) was administered daily on the skin of the back of the patients enrolled. The standard dose was 2 mg/day for 30 to 60 days. Intermediate clinical control was scheduled. The surgical repair was performed by the same senior pediatric surgeon after a one-month interval since the interruption of the PHT.

The clinical charts and surgical reports were reviewed. The following outcomes were considered: PL and GW variation, adverse events to transdermal PHT, defined as signs or symptoms that required stopping the protocol, such as painful erection, scrotal hyperpigmentation, pubic hair, or skin irritation. The rate of surgical complications, such as urethral fistula and/or wound dehiscence, was also reported.

Statistical analysis was performed as follows: The qualitative variables were described as frequency (%). The continuous variables were reported as mean and standard deviation (SD). The Cohen’s effect size (d) was also calculated for PL and GW variation to assess significant differences between PL and GW before and after the PHT. Data were analyzed using SPSSv10.0 (SPSS Inc., Chicago, IL, USA).

## 3. Results

During the study period, 12 patients met the inclusion criteria. In one case (8.3%), the legal guardians refused to give consent. Another family did not receive the topic testosterone gel. The overall participation rate was 83%.

Table 1 summarizes the clinical history, characteristics, and endocrinological features of the ten patients enrolled. The standard karyotype together with the AMH levels helped in the assignment of the male sex rearing. No cases of hypogonadotropic hypogonadism or adrenal insufficiency were detected. No endocrine contraindications to transdermal PHT were found. The external genitalia score (EGS) was between 7.5 and 10.5 [13].

**Figure 1:**
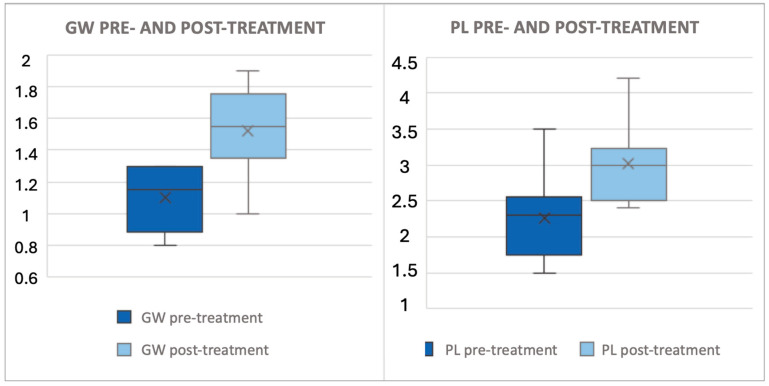
This figure illustrates the difference in glans width and penile length before and after PHT. All data are expressed in centimeters. The x denotes the mean value, while the box plot delineates the median and interquartile range (IQR). The PL increased by 0.76 (±0.27) cm (+37%) and the GW increased by 0.42 (±0.26) cm (+40%), as outlined in Table 2.

The mean age at surgery was 2.7 (±1.6) years. Six of the patients (60%) had proximal hypospadias. Transdermal PHT was applied for a mean of 43 (±15) days on average. The mean interval before surgery was 52 (±23) days. No adverse events associated with transdermal PHT were reported. The penile tissue response, such as PL and GW variation, is described in Table 2 and graphically represented in Figure 1. The Cohen’s effect size can be considered clinically relevant (d = 1.647; d = 1.305, respectively).

Five patients (50%) required a staged surgical repair. The TIPU technique was preferred for single-stage repair (five cases), while the Duckett technique (one case), the Bracka procedure (two cases) and the TIPU technique (two cases) were performed for staged surgical repair. The mean follow-up was 31.1 (±2.7) months. The overall complication rate was 30%. Urethral fistula was reported in two cases and a glandular dehiscence in one case. All the patients affected by complications suffered from proximal hypospadias.

## 4. Discussion

The population under investigation consisted of young children with severe degrees of hypospadias and an EGS ranging between 7.5 and 10.5. The EGS is a tool that can be used to describe the degree of virilization of the external genitalia, ranging from 0 to 12 (from female to male), and it has been validated for children aged 0 to 24 months [13]. The observed variation in the study population may be due to age differences between subjects, with subjects older than 24 months potentially contributing to the observed variability. Nonetheless, the clinical indicators of decreased virilization, including penis length, glans width, and meatus position, were evident. The transdermal PHT was well-tolerated with good compliance by the children and their caregivers. These preliminary results found a clinically relevant increase in both GW and PL. Although it was not feasible to provide genetic assessment to the entire population, it can be hypothesized that within the cases of hypospadias under consideration, seeing the good response to the PHT, the presence of androgen insensitivity syndrome was negligible. A meta-analysis of Zhao W. et al. aimed to define the prevalence of androgen insensitivity in 306 hypospadias patients treated with preoperative hormone therapy and found a possible prevalence of 7.1% (25 cases did not respond to the preoperative hormonal treatment, showing androgen resistance) [14].

Despite the controversy surrounding the efficacy and the indications of PHT for hypospadias repair [15], this strategy has not been abandoned by surgeons dealing with severe hypospadias. This is due to the fact that, during the surgical procedure, the PHT may provide the surgeons with increased tissue, thus enabling them to perform a safer surgery. However, it is important to determine whether the use of PHT is beneficial only in terms of facilitating surgical repair or whether it can also serve to reduce the incidence of complications. To answer this question, the synergic interaction between the surgeon and pediatric endocrinologist may help identify the cases that could benefit from PHT, aiming to improve surgical outcomes. It is crucial to note that severe hypospadias may be categorized as part of the differential diagnosis of differences in sex development (DSD), with genetic and/or endocrinological causes. The involvement of a pediatric endocrinologist in the management of severe hypospadias, particularly during the initial months of life, is paramount. This temporal window may facilitate the elucidation of endocrinological causes of severe hypospadias and enable effective intervention. A large series of patients with proximal hypospadias exhibited an elevated GW in 70% of cases when the transdermal or intramuscular PHT was administered prior to the second-stage intervention, without any alteration in the incidence of surgical complications or severe adverse effects [16]. Since the reconstruction of a small glans represented a limiting factor for a good outcome [4], the PHT should be offered to selected patients with proximal hypospadias. On the other hand, another study failed to ascertain a safe GW of more than 15 mm [6], possibly due to the characteristics of the participants that were affected by the most severe forms of hypospadias. Nevertheless, in authors’ opinion, this should not be enough to stop the approach with PHT, as every gained millimeter might be extremely helpful to the surgeon.

The topic of PHT has been introduced for the last two decades, and no difference in efficacy with intramuscular administration has been found [17]. However, it might be speculated that transdermal administration presents some innovative aspects: (i) the approach is not as invasive as the intramuscular administration, and (ii) the testosterone gel can be administered on the back skin without any direct manipulation of the genitals. These aspects could positively impact the acceptance and compliance to the therapy. Eventually, a constant hormonal stimulation might be more physiological with potential positive effects on the tissue response. However, this hypothesis requires further studies to be proved.

The rate of complications in our patients was similar to those reported in the literature [18]. The influence of PHT on postoperative complications is another point of discussion. It is well-known that the rate of complications is higher after severe hypospadias repair. Since PHT was usually indicated for these peculiar patients, a selection bias might explain a higher rate of complications after PHT [19].

## 5. Conclusions

Despite the limited sample size, which does not allow us to draw definitive conclusions, the preliminary results shown here seem to highlight the tolerability and efficacy of the transdermal PHT for patients affected by severe hypospadias and highlight the importance of synergic cooperation between pediatric endocrinologists and surgeons.

## 6. Limitations

Our study has major limitations due to the number of cases presented and the lack of a control group. Age at surgery is different from one case to the other. In addition, our results are only based on short-term follow-up, and we do not show long-term functional outcomes or fertility data. We only show severe cases of hypospadias, so the results might be influenced by selection bias. Being a retrospective study, a few data points on hormonal assessment are also missing.

## Figures and Tables

**Table 1:** Anthropometric and laboratory data on patients.

Case	1	2	3	4	5	6	7	8	9	10
**Neonatal carachteristics**	Term age, AGA	Term age, AGA	Late preterm, SGA, IUGR	Term age, SGA, IUGR	Preterm, SGA, IUGR	Term age, AGA	Twin pregnancy, Preterm, AGA	Term age, AGA	Preterm, SGA, IUGR	Term age, AGA
**Age at the** **endo-surgeon assessment** **(years)**	2.11	2.13	1.89	0.24	1.02	4.62	5.3	1.14	3.94	1.33
**Height, cm** **(SDS)**	91 (0.86)	93 (1.46)	82.6 (−1.38)	56 (−2.47)	77.3 (1.77)	98 (−2.12)	104.8 (−1.51)	76 (−0.67)	99 (−0.97)	79.7 (−0.2)
**BMI, Kg/m2** **(SDS)**	15.46 (−0.42)	13.87 (−1.87)	15.24 (−0.47)	16.61 (−0.3)	15.35 (−1.33)	13.3 (−1.63)	14.84 (−0.72)	17.56 (0.7)	13.98 (−1.61)	19.3 (2.02)
**Penile lenght (cm)**	2.5	2.2	1.6	1.4	1.9	2.7	1.8	2.1	3.5	1.5
**Glans width (cm)**	1.3	1.3	0.9	0.5	0.8	0.85	1.3	1.1	1.1	1.1
**Meatus position**	Scrotal	Scrotal	Scrotal	Scrotal	Scrotal	Scrotal	Medial	Medial	Medial	At the penis base
**Testis vol. L/R** **(mL)**	1/1	2/2	1/1	1/1	1/1	1.5/0.5	1.5/1.5	1/1	1/1	2/2
**AGD-AS** **(cm)**	3.2	5		2.4	1.9	0.7		2.2	2.9	2.4
**AGD-AP** **(cm)**	6.9	6.8		5.3	6.2	6.2		7.1	7.6	7.2
**EGS**	8.5	8.5	8	8	7.5	9	9	9.5	10.5	9
**LH** **(mIU/mL)**	0.2	<0.1	<0.1	4.3	0.9	0.2	<0.1	0.2	0.2	0.3
**FSH** **(mIU/mL)**	0.7	1.2	0.4	2.4	1.9	1.2	0.8	0.5	0.4	0.3
**Testosterone** **(ng/mL)**	0.1	0.1	0.1	1.6	0.1	0.1	0.1	0.1	0.1	0.1
**DHT** **(pg/mL)**	18	23	22			14	53	33.8		
**T/DHT**	5.56	4.35	4.54			7.14	1.89	2.96		
**AMH** **(ng/mL)**	39	23.5		61		79	24.2	100	39	
**Androstenedione (ng/mL)**	<0.240	<0.240	0.28	0.29			0.26	<0.240	<0.240	<0.240
**DHEAS** **(microg/mL)**	<0.02	0.04	0.06	0.33			0.08	0.02	0.44	0.02
**17-OHP** **(ng/mL)**	0.3	0.3	0.5	1.7	2.4	0.1	0.4	0.5	0.6	0.9
**ACTH** **(pg/mL)**	13.4	14.5	28.5	62.4		12.6	15.5	42.5	6.4	10.3
**Cortisol** **(microg/dL)**			4.5	7.9	2.4	4.9		7.9	4.7	8.7
**Karyotype**	46, XY	46, XY	46, XY	46, XY	46, XY	46, XY	46, XY			
**Array-CGH**		Normal		Normal	Del (9p24.3)	Normal	Normal			
**Minipuberty assessment**	Age adequate	Age adequate	NA	Age adequate	Age adequate	Age adequate	NA	NA	NA	NA

**Table 2:** Penile tissue response to transdermal preoperative hormonal treatment.

**Glans Width (mm)**					
	*Pre-treatment*	*Post-treatment*	*Increase*	*%Increase*	*Cohen’s effect size (d)*
**Mean**	10.9	15.1	4.2	+40%	1.647
**SD**	2	3	3		
**Penile Length (cm)**					
	*Pre-treatment*	*Post-treatment*	*Increase*	*%Increase*	*Cohen’s effect size (d)*
**Mean**	2.26	3.03	0.76	+37%	1.305
**SD**	0.60	0.58	0.27		

## Data Availability

Data is unavailable due to privacy.
